# Supplementary material for: Integrating psychosocial variables and societal diversity in epidemic models for predicting COVID-19 transmission dynamics
Source: PLOS Digit Health. 2022 Aug 31;1(8):e0000098. doi: 10.1371/journal.pdig.0000098 (PMC9931295; doi:10.1371/journal.pdig.0000098)
Supplement: S1 Text — (DOCX) [file pdig.0000098.s001.docx]

## Supplementary Materials to

## “Integrating psychosocial variables and societal diversity in epidemic models for predicting COVID-19 transmission dynamics”

## by VK Jirsa, S Petkoski, H Wang, M Woodman, J Fousek, C Betsch, L Felgendreff, R Böhm, L Lilleholt, I Zettler, SM Faber, K Shen, AR McIntosh

### Group structure

To obtain a better insight into the nature of the effects of PS coupling, we performed three additional parameter sweeps in Figure S1 along the example of Denmark, separately varying the occurrence of the strictest government intervention (Figure S1A), PS coupling strength (Figure S1B) and strength of group structure (Figure S1C). An implementation for Germany shows the same results. In all three panels the time evolution of the number of susceptible and infected individuals (S and I) is plotted, together with the reproduction number *R*, and the time-course of interventions *J* in the first one, and the affective state $\phi$ in the other two panels. The simulations of Figure S1A were performed by varying the timing of the strictest government interventions from 20 days earlier than it has actually occurred, up to 20 days later. The results show that besides the extreme case of the very late intervention (red), a very early intervention would also lead to a large peak later on, after the eventual relaxation occurs. However, there seems to be a preferable timeslot for interventions, which is around the time of the actual one (green). Besides shifting the peak to allow time for more preparation, these interventions also flatten it more than the two extremes, by avoiding the large initial peak as well as a late second overshoot, keeping the number of infected people over time small, allowing the system to prepare the best available resources. The simulations of Figure 5B were performed as a function of sweeps of γ with no group structure. As the PS coupling strength γ increases, the influence of PS variables upon the mixing behavior becomes stronger, advancing systematically the peak of the second wave of infections to earlier time points and increasing its size. The time courses of S and I show two convergent regimes, towards very small and large values of γ. For S, it expresses itself as increased densities of lines at the blue and red ends of the spectrum. Equivalently, a bimodal organization of I appears in blue for vanishing γ and red for strong PS influence. The organization in two apparent limit cases is a consequence of the interaction of political interventions *J* and PS dynamics. Given a duration of a lock-down, the sensitivity towards the variation of γ is particularly strong in the regimes of γ =0.15-0.7, but very small below or above this window, as the effects accumulate in the high and low-coupling modes (the clustered blue and red lines in *S* and *I*). The value of coupling strength γ is thus an important nation-specific indicator for a recommended lock-down duration, which will vary from country to country for the same intervention strength *J*. Figure S1C shows the cause-effect scenarios due to group structure for Denmark. The strength of PS coupling is set constant to the value estimated from the Danish data, and the heterogeneity of the groups is linearly increased from fully homogeneous ($c_{1}$=$c_{2}$=1, $\Gamma_{1}=\Gamma_{2}=1$) to strongly separated groups ($c_{1}$=1, $c_{2}$=30, $\Gamma_{1}=1,\Gamma_{2}=30$). Differences in vulnerability show large effects upon affective state $\phi_{i}$and subsequently in the number of infected cases per group. Small differences in heterogeneity result in similar infection numbers for both populations; but the larger the group differences, the bigger effects upon both groups, with larger numbers of infections in the vulnerable population. It needs to be stressed that all other parameters are identical and only the group differences are present causing the acceleration and increase of numbers of epidemic stress. This clearly highlights the influence of societal organization with regard to different levels of PS variables (e.g., risk perceptions) on epidemic transmission. The effect of group structure is very pronounced and remains robust across parameter sweeps.

### PS indicators

It is debatable whether the measures we aggregated above capture all aspects of PS variables linked to (perceived) vulnerability and resilience. We assessed this using additional measures of subjective psychological well-being and resilience (collectively PSY/RES, see Table A below for details) that were collected for a subset of the waves (March 24 and 31, April 21, May 26 in the German data, and March 24 and 30, April 6, 14 and 22 in the Danish data). We used PSY/RES measures to relate to the aggregate of AFFECT, POLICY, and TRUST measures (AFFECT aggregate) to first determine the strength of the relation and, second, whether that relation changed across time. We found the higher levels of AFFECT (i.e., more concern with COVID-19) were related to less resilience (Figure 5). Importantly, this relation did not show an appreciable change across waves, suggesting the AFFECT aggregate does relate to psychological well-being and resilience.

### Sample descriptions:

Sample Size by groups from COSMO data from Germany by acquisition wave (week).

|  | Young (18-37 yrs) | Middle (38-55 yrs) | Old (56-87 yrs) |  | Males | Females |
| --- | --- | --- | --- | --- | --- | --- |
| March 3 | 270 | 274 | 278 |  | 425 | 397 |
| March 10 | 250 | 310 | 274 |  | 410 | 424 |
| March 17 | 305 | 307 | 289 |  | 455 | 446 |
| March 24 | 298 | 274 | 377 |  | 491 | 458 |
| March 31 | 279 | 350 | 291 |  | 467 | 453 |
| April 7 | 300 | 332 | 297 |  | 474 | 455 |
| April 14 | 341 | 302 | 317 |  | 473 | 487 |
| April 21 | 275 | 324 | 310 |  | 451 | 458 |
| April 28 | 341 | 301 | 293 |  | 451 | 484 |
| May 5 | 319 | 306 | 274 |  | 462 | 437 |
| May 12 | 309 | 287 | 311 |  | 445 | 462 |
| May 19 | 346 | 280 | 251 |  | 438 | 439 |
| May 26 | 307 | 259 | 261 |  | 400 | 427 |

Sample Size by groups from COSMO data from Denmark by acquisition wave (week).

|  | Young (18-49yrs) | Middle (50-63yrs) | Old (64-92yrs) |  | Males | Females |
| --- | --- | --- | --- | --- | --- | --- |
| March 24 | 198 | 156 | 155 |  | 217 | 292 |
| March 30 | 151 | 133 | 137 |  | 173 | 248 |
| April 8 | 128 | 131 | 141 |  | 166 | 234 |
| April 14 | 161 | 153 | 161 |  | 213 | 262 |
| April 22 | 202 | 165 | 194 |  | 237 | 324 |
| April 28 | 164 | 170 | 205 |  | 228 | 311 |
| May 5 | 170 | 196 | 194 |  | 236 | 324 |
| May 13 | 190 | 171 | 218 |  | 280 | 299 |
| Jun 2 | 207 | 208 | 312 |  | 336 | 391 |
| Jun 16 | 161 | 179 | 201 |  | 246 | 295 |

### PLS

Partial Least Squares (PLS): PLS is a multivariate statistical analysis method similar to canonical correlation analysis. We used it here to first determine if there was a linear combination of PS indicators that differed by group. For this analysis, COSMO data were grouped according to sex for one analysis and into three age groups for the second of about the same sample size. The age bins in the German data, age categories were 18-37, 38-55 and 56-87 years. In Danish data, age categories were 18-49, 50-63 and 64-92 years. Data were then grand mean-centered and decomposed using singular value decomposition (SVD), yielding two matrices of singular vectors (U, V) and a diagonal matrix of singular values (S). The singular vectors in V contain the weights for groups by wave, and U contains the weights for the PS indicators. For each pair of vectors in U and V, the value in S is essentially a covariance indicating the strength of the relationship between the U and V vectors. The statistical significance of the relationship between vectors is assessed using permutation tests where each observation is a randomly reassigned group and the mean-centering the SVD done for the permuted data. The permutation is performed 1000 times to create a distribution of S. For each pair, the corresponding original value of S is compared to the permuted distribution and assigned a probability value based on the number of times a permuted S was greater than or equal to the original value. A complementary resampling assessment is done for the weights in U and V using bootstrap. Here resampling is done with replacement, maintaining the group assignment, but assessing how reliable the parameter estimates in U and V are depending on which subjects are in the sample. The resampling is done 1,000 times, producing a distribution of the parameters in U and V, which are used to estimate confidence intervals or standard errors. The ratio of a singular vector weight by its standard error is termed a “bootstrap ratio” and is similar to a z-score, but interpreted more as indication of parameter stability rather than null hypothesis testing. The latter is done at the level of the singular value using permutation testing.

**Table A. *COSMO* PS and psychological well-being indicators included for analysis.**

| **Variable name** | **Survey question** | **Included in dataset from:** | |
| --- | --- | --- | --- |
|  |  | **Germany** | **Denmark** |
| ***AFFECT (AFF_)*** | ***To me, the novel coronavirus (COVID-19) feels…*** |  |  |
| **DISTANCE** | Close to me (1)  Far away from me (7) | X | X |
| **FEAR** | Terrifying (1)  Not terrifying at all (7) | X | X |
| **HYPE** | Overly covered in the media (1)  Not enough coverage in the media (7) | X | X |
| **SPREAD** | Like it spreads slowly (1)  Like it spreads quickly (7) | X | X |
| **THINK** | Like something I think of all the time (1)  Like something I don’t think about at all (7) | X | X |
| **WORRY** | Like something to worry about (1)  Like something not to worry about (7) | X | X |
| **NOVELTY** | New (1)  Old (7) |  | X |
| **SCIENCE** | Not at all scientifically described (1)  Completely scientifically described (7) |  | X |
| **HELPLESS** | Like something that makes me feel helpless (1)  Like something I can combat with my own actions (7) |  | X |
| ***POLICY (POL_)*** | ***Please indicate the extent to which you disagree (1) or agree (7) with the following statements.*** |  |  |
| **FREEDOM** | It makes sense that the government restricts personal liberty rights to combat the novel coronavirus (COVID-19). | X | X |
| **INTERNET** | The government should restrict access to the Internet and social media to combat the spread of misinformation about the novel coronavirus. | X |  |
| **EXAGGERATE** | I think the measures that are currently being taken are greatly exaggerated. | X | X |
| **SCHOOL** | As a precaution, community facilities such as schools or kindergartens should be closed. | X |  |
| **VACCINE** | If a vaccine against the novel coronavirus (COVID-19) becomes available, I would get it. |  | X |
| **TRAVEL** | It makes sense that the Ministry of Foreign Affairs discourages all non-essential travel. |  | X |
| **TREATMENT GUARANTEES** | It makes sense that guarantees for treatment and examination within the health care system have been suspended by law. |  | X |
| **EPI LAW** | It makes sense that epidemic legislation has been tightened so that the authorities have the right to impose sanctions on individuals they believe might spread infection. |  | X |
| **FORCE TREATMENT** | It makes sense that the authorities have the power to coerce people to compulsory treatment if there is suspicion of infection with the novel coronavirus (COVID-19). |  | X |
| **FORCE VACCINE** | It makes sense that the authorities have the power to force people to get vaccinated against the novel coronavirus (COVID-19). |  | X |
| **FORCE QUARANTINE** | It makes sense that the authorities have the power to force people to quarantine if there is suspicion of infection with the novel coronavirus (COVID-19). |  | X |
| **POLICE FORCE** | It makes sense that the police have the power to use the necessary power to enforce the new legislation adopted in the fight against the novel coronavirus (COVID-19). |  | X |
| **FORBID ACCESS** | It makes sense that the authorities have the possibility to ban access to public institutions, supermarkets and shops, public and private nursing homes and hospitals, as well as the possibility to put restrictions on transportation. |  | X |
| **TRUST** | ***How much trust do you have in the people and organizations below that they are able to handle the new type of coronavirus properly and correctly? (Very little: 1, A lot of trust: 7)*** |  |  |
| **FEDERAL HEALTH** | The Federal Ministry of Health | X |  |
| **HOSPITAL** | Hospitals | X |  |
| **MEDIA** | The media | X |  |
| **RKI** | The Robert Koch Institute for Public Health | X |  |
| **STATE HEALTH** | Your state’s Ministry of Health | X |  |
| **POLICE** | The police |  | X |
| **LOCAL BUSINESS** | Private businesses |  | X |
| **HOSPITALS DOCTORS** | Hospitals and doctors |  | X |
| **GOVERNMENT** | State authorities |  | X |
| **EXPERTS** | Experts (e.g., researchers) |  | X |
| **POLITICIANS** | Politicians |  | X |
| **PSYCHOLOGICAL WELL-BEING** | ***Germany: Below are descriptions of how you may have felt or behaved most recently. Please choose the answer that best suits or complies with your condition during the past week.***    ***Denmark*: Questions concerning your psychological state.** | 1: Not at all or <1 day  2: 1-2 days  3: 3-4 days  4: 5-7 days | 1: Not at all  5: Extremely so |
| **NERVOUS** | I felt nervous, anxious or tense. | X |  |
| **DEPRESSED** | I felt down/depressed. | X |  |
| **LONELY** | I felt lonely. | X |  |
| **HOPEFUL** | I thought about the future with hope. | X |  |
| **STRESSED** | I feel stressed out at the moment |  | X |
| **ISOLATED** | I feel isolated at the moment |  | X |
| **LONELY** | I feel lonely at the moment |  | X |
| **BORED** | I am bored at the moment. |  | X |
| **OPTIMISTIC** | I am very optimistic when I think about the future. |  | X |
| **SATISFIED** | Overall, how satisfied are you with your life at the moment? |  | X |
| **RESILIENCE** | ***Please indicate how much you agree with the following statements. (1: Do not agree at all, 5: Completely agree)*** |  |  |
| **HARD TIMES** | I tend to recover quickly from difficult times. | X | X |
| **EVENTS** | I find it difficult to endure stressful events. | X | X |
| **RECOVERY** | It does not take me a lot of time to recover from a stressful event. | X | X |
| **NORMAL** | It is hard for me to get back to my usual self after something unpleasant has happened. | X | X |
| **MANAGE** | I usually manage difficult times without great difficulty. | X | X |
| **SETBACKS** | I tend to take a long time to recover from setbacks in my life. | X | X |

**S1 Fig.** **Covid-19 spread and cause-effect scenarios under variation of lock-down duration, PS coupling strength and societal heterogeneity.** Parameter sweeps for (A) the government interventions, (B) coupling strength of the PS system, and (C) the group specific parameters. The results for the default parameters are always green. For clarity, the impact of seasonality is not included and only the current interventions are used to better identify the impact of the government interventions and the PS subsystem and its heterogeneity. All simulations are performed for Denmark.

**S2 Fig.** Estimated strengths of Interventions on infectious spread for Germany (same as in Figure 4C).

**S3 Fig. Relationship between subjective psychological well-being and resilience and PS indicators.** First latent variables (both p<0.001) of a multivariate PLS analysis relating psychological well-being (PSY/RES) to the AFFECT aggregate in German (A-B) and Danish (C-D) COSMO data. Pearson correlation coefficients (A and C) show a stable relationship across time. See Table A in S1 Text for a complete list of variables. Bootstrap ratios are singular value weights divided by their standard error and are roughly equivalent to a z-score.

**S4 Fig.** **Age differences in PS indicators.** The dominant latent variable (both p<0.001 by permutation test) of a multivariate PLS analysis of mean change over time of PS indicators in German (A-B) and Danish (C-D) COSMO data by age group. Subjects were grouped into three equally sized bins of age categories. In the German data, age categories were 18-37, 38-55 and 56-87 years. In Danish data, age categories were 18-49, 50-63 and 64-92 years. AFFECT (AFF), POLICY (POL) and TRUST variables were included if data were available from all waves in each of the COSMO datasets. See Table A in S1 Text for a complete list of variables. Error bars: 95% CI.

**S5 Fig.** **Data available for Germany and Denmark from February 15th to early of May 2021.** Estimates and reported daily cases and reproduction number R are presented as time varying means and standard deviations. Data recovered from <https://covid19.healthdata.org/>.
