# Supplementary figures and images for: Integrating psychosocial variables and societal diversity in epidemic models for predicting COVID-19 transmission dynamics
Source: PLOS Digit Health. 2022 Aug 31;1(8):e0000098. doi: 10.1371/journal.pdig.0000098 (PMC9931295; doi:10.1371/journal.pdig.0000098)

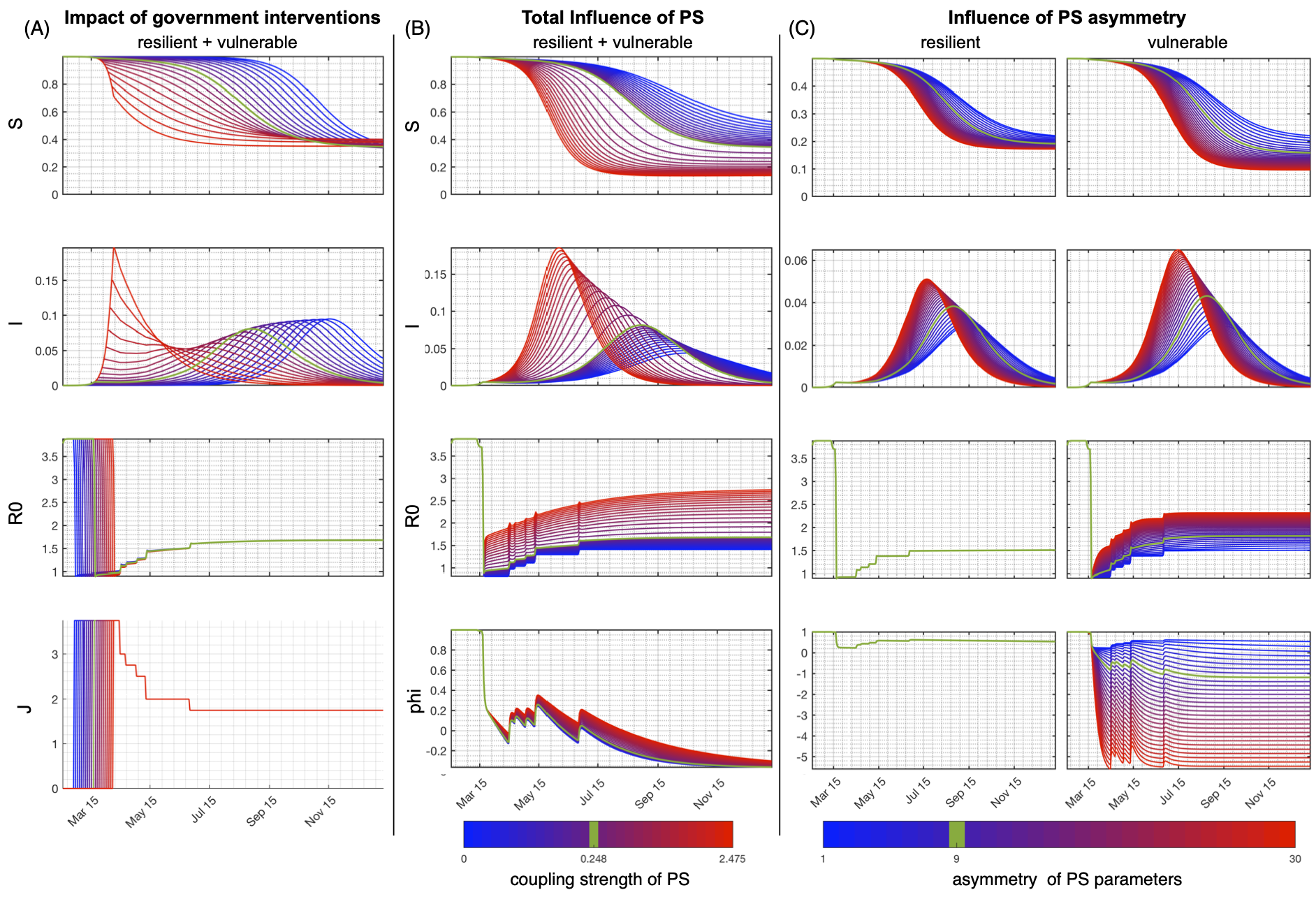

Supplement: S1 Fig — Parameter sweeps for (A) the government interventions, (B) coupling strength of the PS system, and (C) the group specific parameters. The results for the default parameters are always green. For clarity, the impact of seasonality is not included and only the current interventions are used to better identify the impact of the government interventions and the PS subsystem and its heterogeneity. All simulations are performed for Denmark. (TIFF) [file pdig.0000098.s002.tiff]

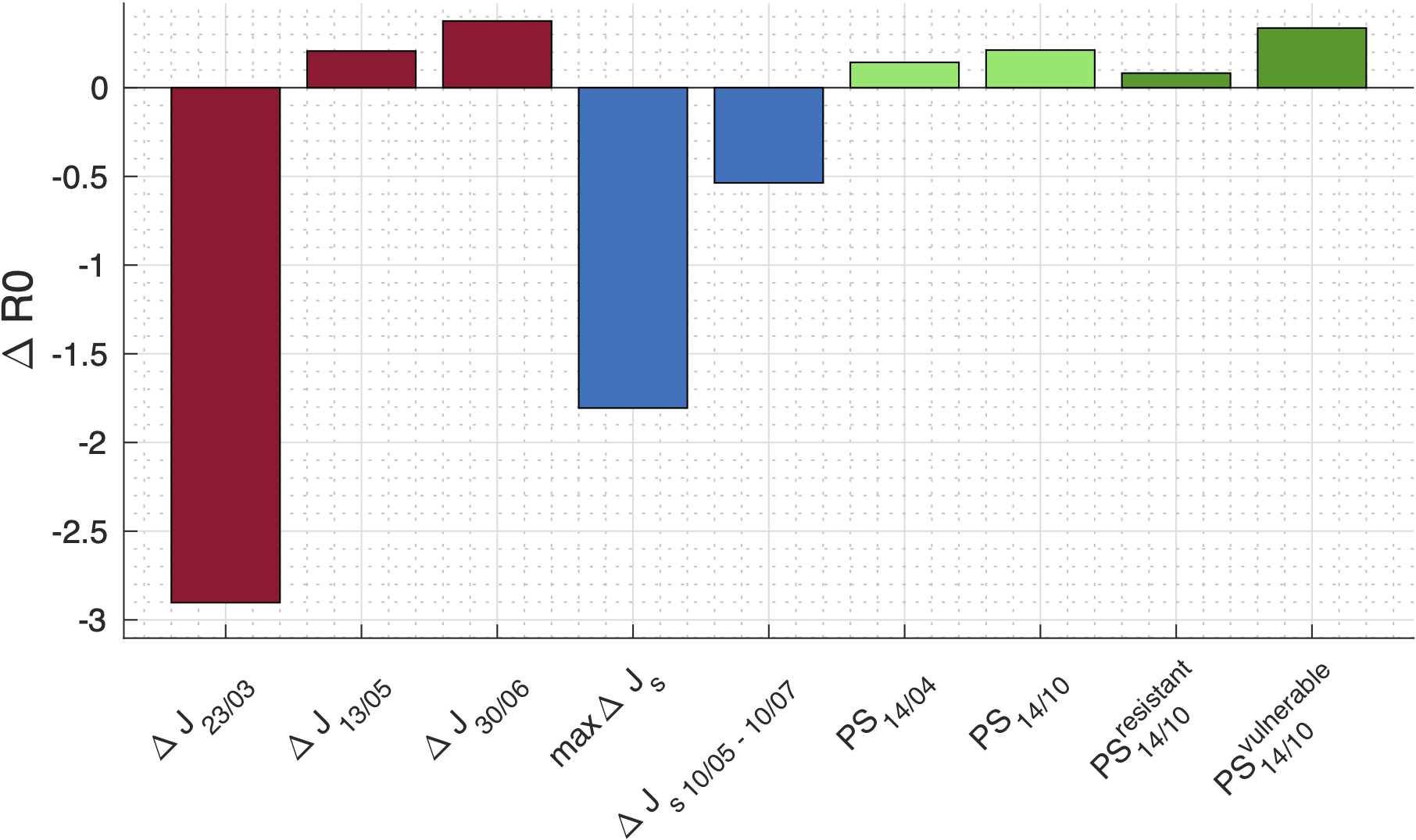

Supplement: S2 Fig — (TIFF) [file pdig.0000098.s003.tiff]

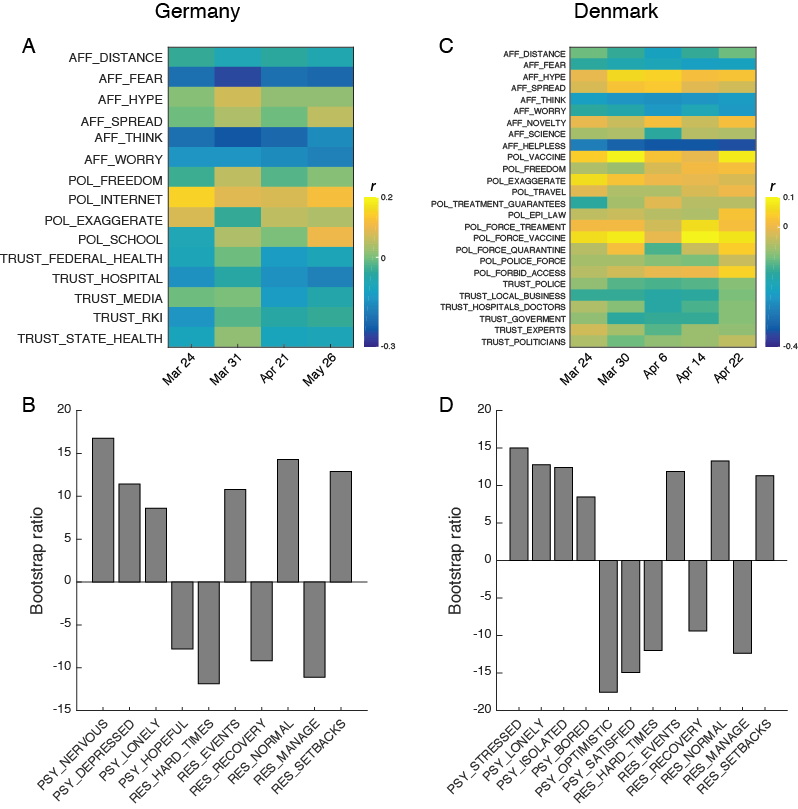

Supplement: S3 Fig — First latent variables (both p<0.001) of a multivariate PLS analysis relating psychological well-being (PSY/RES) to the AFFECT aggregate in German (A-B) and Danish (C-D) COSMO data. Pearson correlation coefficients (A and C) show a stable relationship across time. See Table A in S1 Text for a complete list of variables. Bootstrap ratios are singular value weights divided by their standard error and are roughly equivalent to a z-score. (TIFF) [file pdig.0000098.s004.tiff]

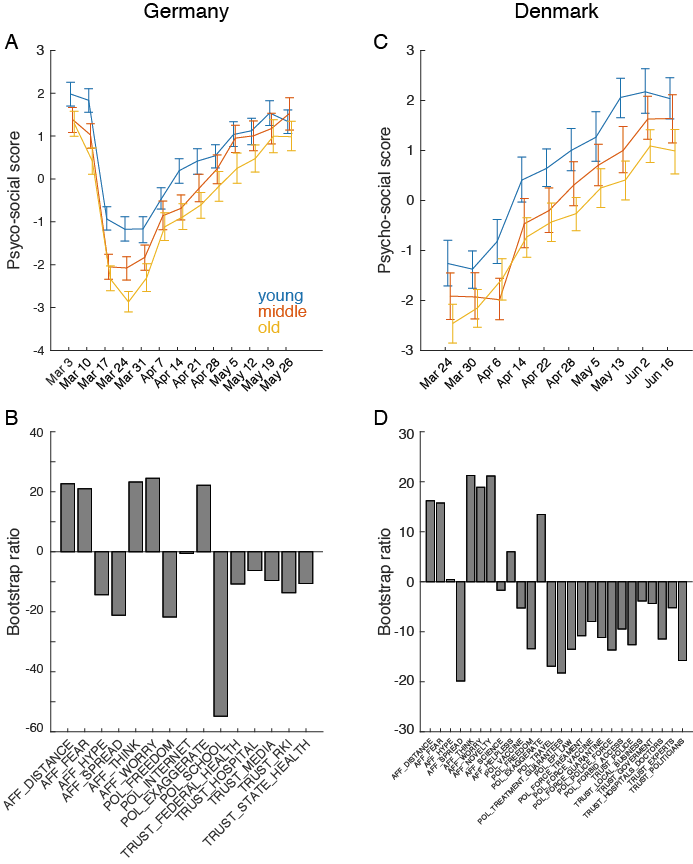

Supplement: S4 Fig — The dominant latent variable (both p<0.001 by permutation test) of a multivariate PLS analysis of mean change over time of PS indicators in German (A-B) and Danish (C-D) COSMO data by age group. Subjects were grouped into three equally sized bins of age categories. In the German data, age categories were 18–37, 38–55 and 56–87 years. In Danish data, age categories were 18–49, 50–63 and 64–92 years. AFFECT (AFF), POLICY (POL) and TRUST variables were included if data were available from all waves in each of the COSMO datasets. See Table A in S1 Text for a complete list of variables. Error bars: 95% CI. (TIFF) [file pdig.0000098.s005.tiff]

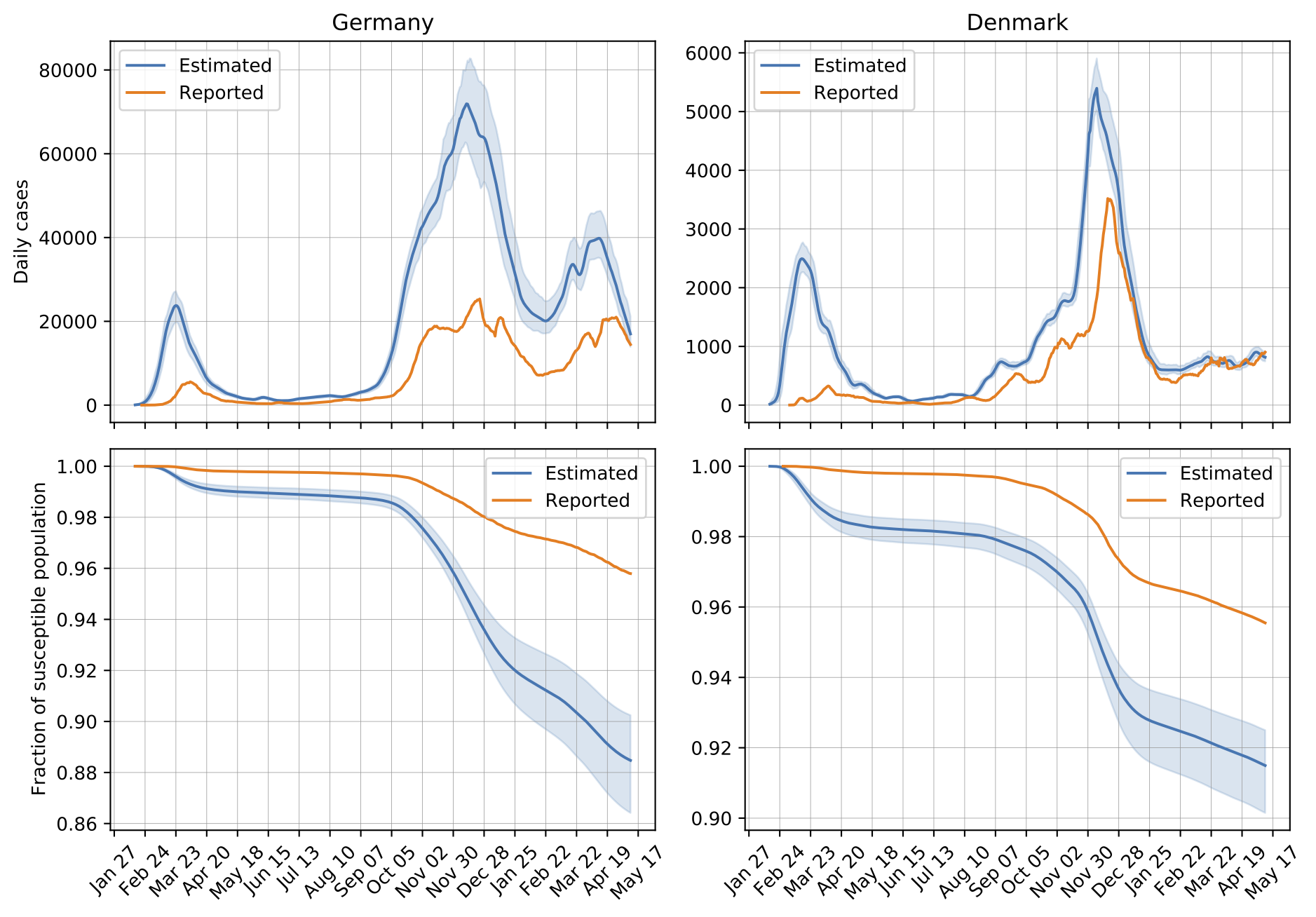

Supplement: S5 Fig — Estimates and reported daily cases and reproduction number R are presented as time varying means and standard deviations. Data recovered from https://covid19.healthdata.org/. (TIFF) [file pdig.0000098.s006.tiff]
